# State of the Blood in Hydrophobia

**Published:** 1842-12

**Authors:** 


*State of the Blood in Hydrophobia.*—The last number of the Austrian *Yahrbücher* contains an interesting case of hydrophobia, with an account of some experiments performed by Professor Berres on the patient's blood.

The blood was dark red in color, and of an oily feel; with the exception of the portion examined in the heart, it furnished very little fibrin and did not coagulate. When examined under the microscope, the globules appeared as perfectly round corpuscules of a dull white color, without any nucleus, and with a few dentated red rays along the edges.

The bicarbonate of potass developed the red colored rays on the corpuscles: the same effect was produced more evidently by a concentrated solution of sulphate of copper, and the nuclei now appeared.

On washing the corpuscles with water, they became of a dull white color, and seemed to pass gradually into a gelatinous, granular mass of molecules.

Concentrated acetic acid turned the corpuscles into molecules. At first, each globule changed into a cluster of small-granules, which soon separated, thus showing the difference between them and the globules of healthy blood.

Under a solution of chlorine the globules remained transparent, but soon changed into a granular mass. When concentrated muriatic acid was mixed with a few drops of the blood, a considerable quantity of the gas was disengaged, and the corpuscles were converted as before into molecules, with a few opaque, contracted globules mixed amongst them.

Treated with concentrated nitric acid the globules became small, round, and opaque; some few were oblong; some pointed at both ends.

Sulphuric ether, iodine, and corrosive sublimate, were also employed with analogous effects. The action of water, chlorine, acetic and muriatic acids, was altogether different from that produced on healthy blood; the three first substances rapidly changing the corpuscles into molecules, and the latter disengaging a remarkable quantity of gas. The peculiarities then observed in the blood in this case may be stated to be—dull white globules, without nuclei; no appearance of the crenated margin; a very quick transition of the corpuscles into molecules, and a tendency to lose their individual character. —*Prov. Med. Jour.*, July 2, 1842.

